# Evaluation of the multifaceted role of social media in cancer patient care

**DOI:** 10.3332/ecancer.2025.2036

**Published:** 2025-11-17

**Authors:** Yousef Roosta, Hero Khezri, Vahid Hoseinpour, Mohamad Jebraeily, Amirhossein Rayegani, Saeed Razavi-Dizaji

**Affiliations:** 1 Department of Internal Medicine, School of Medicine, Imam Khomeini Hospital, Urmia University of Medical Sciences, Urmia 5715789397, Iran; 2 Department of Health Information Technology, School of Allied Medical Sciences, Urmia University of Medical Sciences, Urmia 5756115198, Iran; 3 Department of Emergency Medicine, School of Medicine, Imam Khomeini Hospital, Urmia University of Medical Sciences, Urmia 5714783734, Iran; 4 Department of Radiation Oncologist, Omid Medical and Research Centre, Urmia University of Medical Sciences, Urmia 5716835377, Iran; a https://orcid.org/0000-0002-5476-9718

**Keywords:** palliative care, supportive care, social networking, social media, cancer survivors, patient care management, HBM

## Abstract

**Purpose:**

Evaluating cancer patients’ social media use is crucial for understanding their preferences, needs and health behaviours. This study examined social media use in health management and analysed influencing factors using the Health Belief Model (HBM).

**Methods:**

A descriptive-analytic study was conducted in 2024 at hospitals affiliated with Urmia University of Medical Sciences. A total of 204 cancer patients who actively used social media participated. Data were collected using a structured and validated questionnaire. Descriptive statistics and regression analyses (SPSS v16) were applied to examine HBM constructs and their predictors.

**Results:**

The mean age of participants was 54 years; 54% were male and 46% female. Overall, 38% used social media for healthcare purposes. Perceptions across the HBM constructs were moderate to high. Cues to action had the highest mean score at 3.19 standard deviation (SD = 0.546), followed by perceived benefits (*M* = 3.13, SD = 0.429) and self-efficacy (*M* = 3.11, SD = 0.677). Age significantly negatively predicted self-efficacy (*B* = −0.223, *β* = −0.474, *p* < 0.001), perceived benefits (*B* = −0.144, *β* = −0.485, *p* < 0.001) and cues to action (*B* = −0.112, *β* = −0.296, *p* = 0.001).

**Conclusion:**

The findings highlight the multifaceted role of social networks in cancer patient healthcare. Moderate HBM scores indicate the need for tailored digital interventions to strengthen perceived benefits, self-efficacy and responsiveness to cues to action, ultimately fostering patient-centred care and informed health decisions.

## Background

Enhancing the overall quality of life for cancer patients and survivors has become a critical component of comprehensive treatment plans [1]. Despite improvements in prognoses, cancer survivors often experience long-term consequences of diagnosis and treatment.

These consequences may include physical and psychological challenges such as cancer-related fatigue, anxiety, depression, physical disorders and social difficulties, which can negatively affect their quality of life [2–4]. Access to information about cancer and evidence-based interventions can help alleviate fear and anxiety [5]. To meet their informational and supportive needs, cancer patients use various resources [6], with seeking information and emotional support being common strategies to reduce uncertainty and enhance feelings of control [7]. There is a growing trend to rely on the internet as the primary source of health information, rather than other sources such as family, friends, colleagues, healthcare professionals and traditional networks [5, 8]. Social networking platforms are emerging as significant sources of scientific information, encompassing news, technical discussions and educational tools [9]. Social networks provide tools that facilitate interactions among people, enabling the creation, sharing and exchange of health-related information, including experiences from patients and healthcare professionals, within virtual communities and networks [10, 11]. Platforms such as Facebook, Instagram, Twitter, Snapchat, WeChat, YouTube and WhatsApp are often preferred sources of health information [12]. Social networking platforms are attractive because of their easy accessibility, free usage and potential for anonymity, which enables patients to discuss sensitive conditions more openly and encounter fewer barriers than in face-to-face or non-virtual communities [11]. Primary reasons for using health-related social media include obtaining information about diagnosed illnesses, receiving advice from patients with similar conditions, gaining social support and communicating with healthcare providers [13]. The global accessibility of social media allows numerous patient experiences and recommendations to be shared, providing patients with multiple opportunities to find a supportive community that caters to their needs [11]. Given the increasing use of social media as a health resource, it is important to develop a deeper understanding of how cancer patients use cancer-related social media and their opinions on the benefits and effectiveness of such platforms as sources of support [14]. While global trends indicate increasing use of social media for health purposes, cancer patients in Urmia face distinct contextual challenges that may hinder the platforms’ effectiveness. These challenges include limited engagement by healthcare providers in digital communication, persistent dependence on traditional service delivery methods and insufficient responsiveness to patients’ informational and emotional needs through online channels. Although patients and healthcare organisations recognise social media as useful tools for streamlining care and improving support access, these benefits remain underutilised due to limited two-way interactions. The local barriers above highlight the importance of examining specific usage patterns and limitations related to the use of social media in this population. This study aimed to gain a deeper understanding of this gap by investigating the extent and manner of social media use among cancer patients in healthcare. To achieve this, the Health Belief Model (HBM) was used. This model is one of the well-established frameworks in health sciences that examines patients’ perceptions of the benefits and barriers of health-related behaviours, their belief in their own abilities (self-efficacy) and their responsiveness to cues to action (Cues to Action), helping researchers better understand and predict behaviours related to digital health [15]. The findings of this study help to better understand the informational and supportive needs of patients. The results can serve as a foundation for developing effective strategies to enhance the quality of healthcare and create more efficient supportive solutions in the digital space. Ultimately, this study seeks to illuminate the vital role of social media in promoting the psychological, social and healthcare well-being of cancer patients.

## Methods and materials

### Study design and setting

This research is a descriptive-analytical study conducted in 2024 at the hospitals of Urmia University of Medical Sciences (UUoMS). Cancer patients visiting the hospitals were selected as the study population. UUoMS was chosen due to its significant achievements in utilising modern communication technologies, including the adoption and use of social media in the health sector. A total of 204 patients who used social media were selected as the research sample using a systematic sampling method. In this approach, every nth patient who met the inclusion criteria was approached for participation. The inclusion criteria for the study were: a histopathological diagnosis of cancer, hospitalisation at UUoMS and the use of social media by the patient. Patients who did not use social media or for whom a cancer diagnosis had not been confirmed were excluded from the study. Data were collected through a questionnaire during face-to-face interviews.

### Data collection

The questionnaire, serving as the data collection tool, was developed by the researchers based on a literature review of reputable databases such as PubMed and Scopus, using keywords related to social media and cancer. Additionally, it was developed through a brainstorming session. The session involved professors from various fields: hematology and oncology, health information management, medical informatics, emergency medicine, nursing and social work. These professors were selected through purposive sampling based on their prior research on social media and other technologies in healthcare or cancer. The session was facilitated by a chairperson and one writer and lasted approximately 60 minutes. At the beginning, participants were assured that the information they provided would be kept strictly confidential. To uphold ethical standards, informed consent forms were distributed and permission for audio recording was obtained. Four questions were posed during this session, which involved 10 participants. The questions included: How can social media assist cancer patients in various aspects? What are the self-care or self-management behaviours of cancer patients? How can social media be utilised by patients for consultation regarding their health and treatment options? What are the psychological impacts of social media interactions on cancer patients?

The data obtained from the literature review and brainstorming session were carefully examined and final decisions were made based on essential elements for designing the questions. A preliminary questionnaire was developed and a validation form was prepared to assess its validity, considering three aspects: relevance, clarity and necessity for each question. The validation forms were distributed among 10 specialists in hematology and oncology, nursing, health information management and medical informatics. The Content Validity Index and Content Validity Ratio were calculated for each item. To determine reliability, the questionnaire was distributed among 20 cancer patients and internal consistency between the items was measured, resulting in a Cronbach’s alpha of 0.98.

**Figure 1.**
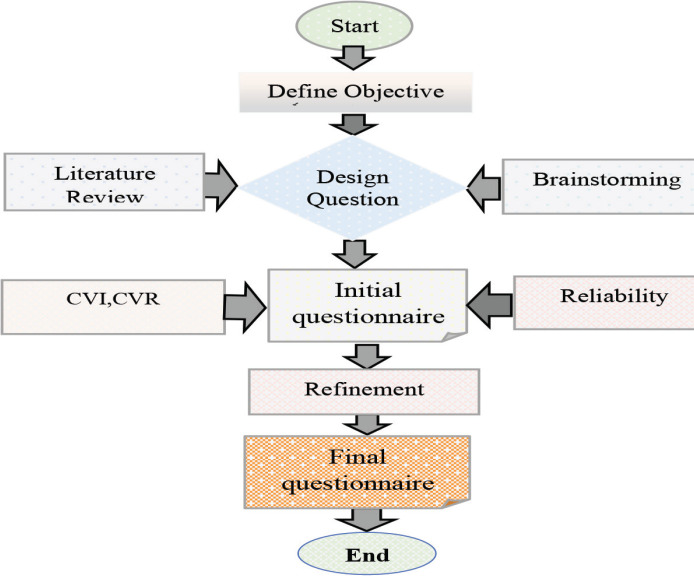
Methodological framework for questionnaire development and validation. Figure methodological framework for developing the questionnaire, including stages of literature review, focus group discussions, initial draft preparation, content validity assessment and reliability testing.

The final questionnaire included 10 variables and 47 questions, comprising demographic information (8 items), social media usage (2 items), Self-care (5 items), Self-management (5 items), Emotional support (4 items), Psychological support (4 items), Information enhancement about cancer (5 items), Communication with the care team (5 items), Social support (5 items) and Mood enhancement (4 items). Respondents’ opinions on self-care, emotional support, information enhancement and interaction with healthcare providers, social support and the usefulness of social media were assessed using a 5-point Likert scale ranging from ‘strongly disagree’ to ‘strongly agree.’ The results were ultimately reported as descriptive statistics. For the purpose of clear data presentation and interpretation, we created a three-level categorisation for the Likert scale responses. The ‘Strongly Agree’ and ‘Agree’ options were combined into a ‘Positive’ category, while ‘Strongly Disagree’ and ‘Disagree’ responses were grouped into a ‘Negative’ category. ‘Neutral’ responses were kept as a distinct, separate category (Figure 1).

The initial design of the questionnaire focused on exploring social media usage patterns in cancer care. However, to provide a more robust theoretical framework for data interpretation, the domains were revised and adapted based on the HBM. It is important to note that this conceptual adaptation did not alter the original questionnaire items, and the questions were categorised solely according to the three domains relevant to the current study’s questionnaire. To assess the internal consistency of the revised framework, Cronbach’s alpha coefficients were calculated for each of the three HBM domains. The results demonstrated strong internal consistency across the board: Perceived benefits (22 items, *α* = 0.84), Cues to action (10 items, *α* = 0.81) and perceived self-efficacy for preventive behaviours and social media use (5 items, α = 0.75). All these coefficients exceed the widely accepted threshold of 0.70, confirming the reliability of the scales within the context of this study.

Data were analysed using SPSS statistical software (version 16). The normality of the data distribution was assessed using descriptive indicators, including the ratio of the standard deviation to the mean (SD/Mean), skewness and kurtosis. Skewness and kurtosis values within the range of ±1, together with an SD/Mean ratio of less than 0.5, were considered indicative of approximate normality. Consequently, multiple linear regression analyses were employed to examine the relationships between the independent variables and the dependent constructs. A statistical significance threshold of *p* <0.05 was applied in all tests.

### Ethical considerations

This study followed ethical guidelines for anonymous survey-based research (approval code: IR.UMSU.HIMAM.REC.1403.104 from the Ethics Council of UUoMS). No identifiable information was collected in the questionnaires, interviews or datasets. All participants provided verbal informed consent prior to completing the questionnaire and interview, after being assured of confidentiality and voluntary participation. All authors certify that the data were processed anonymously in full compliance with ethical research standards.

## Results

### Participant characteristics

The mean age of the participants was 54 years, ranging from 17 to 71 years. Males comprised 54% of the sample (*n* = 111). A substantial portion of the participants (*n* = 160, 78%) were born in urban areas. More than half of the participants (56%) had an education level below a high school diploma. The majority were married (*n* = 171, 84%) and 40% were self-employed (*n* = 82). Most participants had been living with cancer for less than 1 year (*n* = 87, 43%) and 18.6% were diagnosed with breast cancer (*n* = 38).

According to self-reported data, the most frequently used social media were Instagram (64 participants, 31%), WhatsApp (50 participants, 25%) and Telegram (35 participants, 17%). The reported daily duration of social media use was as follows: less than 1 hour (82 participants, 40%), 1–2 hours (64 participants, 31%), 2–3 hours (36 participants, 18%) and more than 3 hours (22 participants, 10%).

### Social media usage patterns

**Figure 2.**
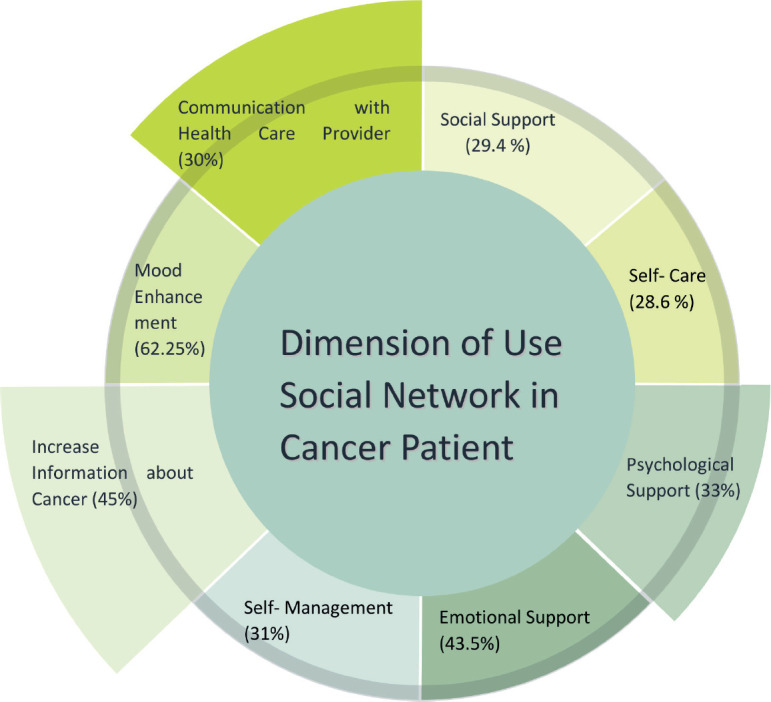
The multifaceted role of social media in cancer care: a visual representation of eight key dimensions. Figure this schematic illustrates eight critical dimensions of social media utilisation in health care for cancer patients. The identified dimensions include: enhanced health information access, psychological support, improved patient morale and motivation, emotional support, self-management, self-care, facilitation of patient-provider communication and social support.

Overall, 38% of cancer patients reported using social media for their healthcare needs. The dimensions of social media usage, ranked by reported frequency, were: mood enhancement (62.25%), information acquisition about cancer (45%), emotional support (43.5%) and psychological support (33%), communication with the medical team (30%), self-management (31%), social support (29%) and self-care (28.6%) (Figure 2).

In the following section, the questionnaire items are presented in order of the highest and lowest percentages across various dimensions, based on participants’ responses in the study.

According to Table 1, the highest percentage (66%) was for the statement ‘Watching funny jokes on social media helps them laugh during treatment.’ The lowest percentage (59%) was for the statement ‘Reading witty or amusing posts on social media makes me laugh during treatment.’

Information acquisition about cancer was identified as the second most common use of social media among participants. The highest percentages (52%) corresponded to ‘I watch and utilise educational videos related to cancer that are shared on social media.’ The lowest percentage (28%) was for ‘I use medical and cancer education applications to enhance my understanding of the disease.’

In the area of emotional support, the highest percentage (49%) was for ‘success stories shared by other patients on social media provide me hope and motivation.’ The lowest percentage (38%) was for ‘participating in group activities on social media increases their sense of purpose and motivation.’

Regarding psychological support, the highest percentage related to psychological support (36%) was for ‘Sharing my cancer experiences on social media enhances my sense of comfort and connection with others.’ The lowest percentage (29%) was for ‘Using social media for emotional support includes expressing feelings about their illness through these platforms.’

In terms of self-management, the highest percentage (39%) was for ‘Social media helps reduce my stress and negative emotions related to cancer,’ while the lowest percentage (20%) corresponded to ‘Social media offers effective strategies for pain management.’

Regarding social support, the highest percentage (34%) was for ‘I follow the channels of cancer patient support associations and charitable organisations on social media and benefit from their support,’ while the lowest percentage (25%) was for ‘I receive support from social workers who inform me about insurance benefits through social media.’

In terms of communication with the medical team, the highest percentage (36%) was for ‘Specialists respond to my questions about my illness, including treatment options, side effects and health tips, through social media,’ while the lowest percentage (23%) was for ‘I participate in scheduled educational programs, such as live sessions and stories, organised by specialists on social media.’

Finally, concerning self-care, the highest percentage (35%) was for ‘The most common practice of using social media to maintain a balanced diet and choose healthy foods.’ The lowest percentage (22%) was ‘I stay informed about potential side effects of treatments and medications’ (22%).

### Descriptive statistics of HBM constructs

As presented in Table 2, the descriptive statistics for the HBM constructs of self-efficacy, cues to action and perceived benefits were analysed for a sample of 204 participants, with findings indicating a moderate level of perception across all three constructs. The mean score for self-efficacy was 3.11 (SD = 0.677), which suggests a moderate level of self-confidence but with the highest variability among responses. Cues to action had the highest mean score at 3.19 (SD = 0.546), indicating that participants generally perceive a moderate level of motivation. Lastly, perceived benefits showed a mean score of 3.13 (SD = 0.429), with the lowest standard deviation, which points to a more consistent perception of benefits among the participants. Overall, the results consistently show a moderate level of engagement with the study’s constructs within the sampled population, with no extreme scores observed in any dimension.

### Predictors of HBM constructs related to social media use in cancer care

Table 3 shows the predictors of HBM constructs from demographic variables. Linear regression analyses were conducted to examine the predictors of self-efficacy, perceived benefits and cues to action. Age was a significant negative predictor across all three models, indicating that higher age was associated with lower levels of self-efficacy (*B* = −0.223, *β* = −0.474, *p* <0.001), perceived benefits (*B* = −0.144, *β* = −0.485, *p* <0.001) and cues to action (*B* = −0.112, β = −0.296, *p* = 0.001). Educational status was a significant negative predictor of perceived benefits (*B* = −0.052, *β* = −0.223, *p* < 0.001) and was marginally associated with cues to action (*B* = −0.037, *β* = −0.126, *p* = 0.068), suggesting that higher education may be linked to slightly lower levels of cues to action. Gender was marginally associated with cues to action (*B* = −0.143, *β* = −0.131, *p* = 0.077); this indicates that women reported higher cues to action than men. Other demographic and clinical variables, including marital status, place of residence, duration of social media use, job and type of cancer, did not show statistically significant associations with the HBM constructs.

## Discussion

The present study›s findings are comprehensively discussed from a dual perspective to provide a robust analysis of social media›s role in cancer patient care. First, we delve into the dimensions of social media use, detailing the specific applications from mood enhancement to self-care that participants employ for health management. Subsequently, we analyse these findings through the lens of the HBM, examining key constructs such as perceived benefits, self-efficacy and cues to action. This integrated approach allows for a nuanced understanding of both the practical uses of social media and the underlying psychological factors that influence its adoption among cancer patients.

### Dimensions of social media use in cancer patient health

Overall, based on participant responses in this study, the use of social media in the health care of cancer patients has been reported at 38%. In a study conducted by Bandar titled ‘The use of the internet and social media among cancer patients,’ the rate of social media usage for obtaining information and support related to cancer was found to be 31% [14]. These findings are somewhat consistent with the overall results of our study.

In the current study, cancer patients have utilised social media in eight different dimensions for their health management, which we will examine below.

One of the most significant applications of social media in the health management of cancer patients is the enhancement of their mood enhancement through laughter therapy. This includes therapeutic interventions aimed at eliciting humorous experiences, which have positive health, physiological and psychological outcomes [16]. Additionally, humour serves as a psychological defence mechanism in cancer care, playing a vital role in allowing individuals to manage their anxiety and stress constructively and coherently [17].

The second application of social media in this context is the acquisition of disease-related information. Cancer patients tend to seek up-to-date information regarding various aspects of their illness, including symptoms, diagnosis, treatment options, side effects of different therapies and the impact of cancer on their lives [18]. Physicians should also assist the public by supervising and disseminating evidence-based content through social media platforms, leveraging their expertise to create and validate such content [19].

The third use of social media in cancer patient healthcare is emotional support. Utilising social media as a new communication channel can significantly enhance mental health by facilitating social interactions and maintaining relationships [20]. Patients can share insights on managing emotional and physical stressors and benefit from shared strategies for coping [21]. Sharing emotional challenges and feelings with others facing similar issues exemplifies emotional support [22, 23].

Psychological support is the fourth application of social media among cancer patients. Psychological resilience, defined as an intrinsic resource, refers to the ability to adapt effectively in the face of adversity [24]. According to the resilience framework, this trait can be activated by external factors, enabling individuals to recover swiftly and successfully from traumatic events, ultimately leading to positive mental health outcomes [25].

Self-management is the fifth dimension of social media use in the health management of cancer patients. Various studies have defined self-management as improving patients’ ability to better control their conditions. These studies indicate that feeling more informed helps patients make better decisions, thereby enhancing their perceived self-management and control over their circumstances [12, 26].

Communication with healthcare providers is another aspect of social media use in cancer patient care. The exchange of medical information with healthcare professionals via social media has increased. This expansion presents a unique opportunity for promoting health through social media [27]. Additionally, a growing minority of physicians are using social networks for direct communication with patients [28]. Social media can also be utilised for scheduling appointments, reporting test results and writing prescriptions [29].

Social support represents the seventh dimension of social media use in health care cancer patients. Charitable organisations significantly leverage social media [30]. Social media can serve as a tool for fundraising and engaging with stakeholders [31].

The final dimension of social media use is self-care. Social media play a crucial role in the self-care of cancer patients, with self-care education potentially enhancing their quality of life and serving as a complementary approach during treatment, particularly during chemotherapy. Self-care programs offer digital tools that empower patients [32]. Furthermore, these platforms can influence healthy lifestyle choices and adherence to treatment regimens [33]. For example, sending two-way text messages can show a 20% improvement in medication adherence [34].

### HBM constructs in social media use for cancer patient health

While these eight dimensions illustrate the practical applications of social media in cancer care, understanding patients’ engagement with these platforms requires a psychological perspective. The HBM provides a useful framework for interpreting patients’ motivations and behaviours regarding social media use. Descriptive analyses indicated that participants’ perceptions of social media use for cancer care were at a moderate level across the three HBM constructs—perceived benefits, self-efficacy and cues to action—highlighting the importance of these key dimensions. Multiple linear regression analyses showed that age was a significant negative predictor for all three HBM constructs, meaning that higher age was associated with lower levels of self-efficacy, perceived benefits from social media use and responsiveness to cues to action. This may reflect less experience with digital technologies and social media or a more cautious attitude toward online health information, resulting in older patients being less likely to trust or use online resources for health management. Educational status was significantly negatively associated with perceived benefits and marginally with cues to action, indicating that individuals with higher education perceived fewer benefits from using social media for health management. This may be explained by higher expectations and more critical appraisal of online information, as more educated individuals may scrutinise social media content more carefully, resulting in a lower perceived benefit. Furthermore, gender was marginally associated with cues to action, with women reporting higher levels than men. This suggests that women are more likely than men to follow and respond to health-related information and messages on social media, actively using online opportunities for health management. Other demographic and clinical variables, including marital status, place of residence, duration of social media use, job and type of cancer, were not significant predictors of any HBM constructs. Overall, these findings indicate that age, educational status and gender play important roles in determining self-efficacy, perceived benefits and responsiveness to cues to action and should be considered in designing digital health interventions. This study complements the research conducted by Issaka *et al* [5]; while their study focused on source credibility and message characteristics in shaping cancer prevention behaviours [35], the present study emphasises individual patient characteristics, such as age, gender and educational level, and HBM constructs in relation to social media engagement. Both studies highlight the importance of psychological factors in health-related behaviours, but from different perspectives: the individual level in the present study versus the message-source level in Issaka *et al* [35]’s research. The findings provide a foundation for designing personalised digital interventions that not only optimise message and source features but also strengthen the psychological constructs influencing patient behaviour. Bak *et al* [36] also employed an HBM-based deep learning approach to predict and analyse public health beliefs regarding breast cancer and screening behaviours using social media data. Their results indicated that public health perceptions were sensitive to political factors, sociological influences, psychological support and environmental conditions, such as the COVID-19 pandemic [36]. In comparison, while Bak *et al* [36]’s study primarily examined macro-level trends and population-wide shifts in health beliefs, the present study focuses on individual-level factors, including age, gender and educational status, in relation to social media use for cancer care. Thus, the current research complements Bak *et al* [36]’s work by providing a more detailed understanding of how personal and disease-specific characteristics influence perceptions and engagement with health information on social media, offering practical insights for designing targeted and psychologically informed interventions.

## Conclusion

This study underscores the multifaceted and critical role of social media in the health management of cancer patients, demonstrating that these platforms can function beyond mere informational tools and provide opportunities for psychological empowerment and enhanced patient engagement. Analysis based on the HBM indicated that perceived benefits, self-efficacy and cues to action are key factors influencing patients’ interactions with social media for health purposes, emphasising the need for personalised digital interventions tailored to individual and contextual patient characteristics. For healthcare professionals and policymakers, these findings highlight the strategic importance of integrating social media into cancer care programs, ensuring that the content provided is evidence-based, accessible and responsive to patients’ specific needs. Accordingly, future research should focus on optimising these platforms, including the development of targeted educational interventions that, taking into account patients’ demographic and personal characteristics, strengthen HBM constructs, facilitate social support and empower patients to actively manage their own health.

## Conflicts of interest

The authors declared no potential conflicts of interest with respect to the research, authorship and/or publication of this article.

## Informed consent

Different ethical aspects of the present research were approved by the Ethics Council of Urmia University of Medical Sciences (IR.UMSU.HIMAM.REC.1403.104). The study adhered to ‘ethical guidelines for anonymous survey-based research.’

## Author contributions

Khezri and Roosta: Research design, overall supervision, methodology, literature review, participation in meetings, manuscript writing, questionnaire design and data collection collaboration.

Hosseinpour and Jebraily: Meeting participation, study coordination, data collection, manuscript editing, collaborative literature review, questionnaire design contribution, data analysis and manuscript editing.

Raigani and Razavi Dizaji: Data collection, meeting participation, questionnaire design contribution and manuscript editing.

## Figures and Tables

**Table 1.** Cancer patient perspectives on dimensions of social networks use in healthcare of cancer patients.

	Strongly agree/agree *N* (%)	Don’t agree nor disagree *N* (%)	Stronglydisagree/Disagree *N* (%)
Perceived benefits			
Enjoyable and lighthearted stories or short clips on social media help me maintain a positive mood during treatment (*Mood Enhancement*)	123 (60%)	49 (24%)	32 (16%)
Upbeat songs and music on social media positively influence my spirits throughout treatment (*Mood Enhancement*)	130 (64%)	40 (20%)	34 (17%)
Watching funny jokes on social media helps them laugh during treatment (*Mood Enhancement*)	135 (66%)	32 (16%)	37 (18%)
Reading witty or amusing posts on social media makes me laugh during treatment (*Mood Enhancement*)	121 (59%)	52 (26%)	31 (15%)
Health recommendations obtained from social media assist me in prioritising my well-being (*Self-Management*)	76 (37%)	57 (28%)	71 (35%)
Social media helps reduce my stress and negative emotions related to cancer (*Self-Management*)	78 (39%)	55 (29%)	68 (33%)
Information from social media supports me in ceasing harmful behaviours, such as smoking or drinking (*Self-Management*)	67 (33%)	64 (31%)	73 (36%)
Social media offers effective strategies for pain management (*Self-Management*)	41 (20%)	71 (35%)	92 (45%)
Social media assists me in managing my medications and understanding their interactions (*Self-Care*)	53 (26%)	69(34%)	82 (40%)
Tips obtained from social media improve my sleep quality (*Self-Care*)	68 (33%)	45 (22%)	91 (45%)
I stay informed about potential side effects of treatments and medications (*Self-Care*)	45 (22%)	63 (31%)	96 (47%)
The most common practice of using social networks to maintain a balanced diet and choose healthy foods (*Self-Care*)	71 (35%)	56 (28%)	77 (38%)
Social media provides exercise programs and motivation tailored to my health condition (*Self-Care*)	55 (27%)	60 (29%)	89 (44%)
I read and share educational articles posted on social media platforms, such as Facebook and WhatsApp (*Increased Information about Cancer*)	100 (49%)	71 (35%)	33 (16%)
I watch and utilise educational videos related to cancer that are shared on social media (*Increased Information about Cancer*)	106 (52%)	66 (32%)	32 (16%)
I access information and resources provided by international cancer associations and organisations (*Increased Information about Cancer*)	94 (46%)	75 (37%)	35 (17%)
I use medical and cancer education applications to enhance my understanding of the disease (*Increased Information about Cancer)*	57 (28%)	108 (53%)	39 (19%)
Social media has simplified the coordination of visits and appointment bookings with my healthcare providers (*Communication with Healthcare Providers*)	61 (30%)	59 (29%)	84 (41%)
I follow the channels of cancer patient support associations and charitable organisations on social networks and benefit from their support (*Social Support*)	69 (34%)	66 (32%)	69 (34%)
Social networks have helped me discover available support packages and services (*Social Support*)	55 (27%)	109 (53%)	40 (20%)
Social networks assist me in locating medical centres related to my disease (*Social Support*)	61 (30%)	85 (42%)	58 (28%)
I receive support from social workers who inform me about insurance benefits and related matters through social networks (*Social Support*)	52 (25%)	62 (30%)	90 (44%)
Self-efficacy			
Joining social media groups facilitates my connection with like-minded individuals and fosters a sense of belonging) *Psychological Support*).	72 (35%)	72 (35%)	60 (29%)
Sharing my cancer experiences on social networks enhances my sense of comfort and connection with others (*Psychological Support*).	74 (36%)	78 (38%)	52 (26%)
Emotional support from friends and followers on social media enhances my feelings of safety and reassurance (*Psychological Support*).	65 (32%)	73 (36%)	66 (32%)
Using social networks for emotional support includes expressing feelings about their illness through these platforms ) Psychological* Support*).	60 (29%)	85 (42%)	59 (29%)
Social media increases my confidence in practicing relaxation methods such as deep breathing, meditation, and yoga (*Self-Management*).	54 (27%)	82 (40%)	68 (33%)
Cues to action			
Success stories shared by other patients on social network provide me with hope and motivation (Emotional Support)	100 (49%)	69 (34%)	35 (17%)
Participation in group activities on social network enhances my sense of purpose and morale (Emotional Support)	78 (38%)	85 (42%)	41 (20%)
Spiritual practices learned through social network contribute to my feelings of calmness and upliftment (Emotional Support)	94 (46%)	72 (35%)	38 (18%)
Receiving advice and support from others on social network fosters a sense of being valued (Emotional Support)	84 (41%)	80 (39%)	40 (20%)
I am a member of channels and groups that provide cancer-related education, and I actively engage with their content (*Increased Information about Cancer*)	98 (48%)	70 (34%)	36 (18%)
Specialists respond to my questions about my illness, including treatment options, side effects, and health tips, through social media (Communication with Healthcare Providers)	74 (36%)	76 (37%)	54 (27%)
In urgent situations, I seek online advice from my doctor regarding my illness via social media (Communication with Healthcare Providers)	69 (34%)	81 (40%)	54 (26%)
Social media has facilitated effective communication with my healthcare team, including nurses and social workers (Communication with Healthcare Providers)	60 (29%)	73 (36%)	71 (35%)
I participate in scheduled educational programs, such as live sessions and stories, organised by specialists on social networks (Communication with Healthcare Providers)	47 (23%)	75 (37%)	82 (40%)
Social networks provide me with essential information regarding ministerial guidelines for cancer patients (Social Support)	64 (31%)	67 (33%)	73 (36%)

**Table 2.** Descriptive statistics of key study variables (*N* = 204).

Variable	Minimum	Maximum	Mean	Std. deviation
Perceived benefits	2	5	3.11	0.677
Self -efficacy	2	4	3.13	0.429
Cues to action	2	4	3.19	0.546

**Table 3.** Predictors of HBM constructs from demographic variables.

Dependent variable		Unstandardised coefficients		Standardised coefficients	*T*	Sig
		*B*	Std. error	Beta		
Self-efficacy	Age	−0.223	0.038	−0.474	−5.794	<0.001***
	Gender	−0.102	0.096	−0.076	−1.068	0.287
	Marital status	0.171	0.139	0.093	1.224	0.222
	Education level	0.015	0.024	0.041	0.628	0.530
Perceived benefits	Age	−0.144	0.023	−0.485	−6.186	<0.001***
	Gender	−0.081	0.058	−0.094	−1.387	0.167
	Marital status	−0.035	0.085	−0.030	−0.411	0.682
	Education level	−0.052	0.015	−0.223	−3.545	<0.001***
Cues to action	Age	−0.112	0.032	−0.296	−3.462	0.001**
	Gender	−0.143	0.081	−0.131	−1.776	0.077^†^
	Marital status	−0.045	0.117	−0.030	−0.380	0.705
	Education level	−0.037	0.020	−0.126	−1.837	0.068^†^

*B* = Unstandardised regression coefficient; SE = Standard Error; *β* = Standardised regression coefficient

** *p* < 0.01; *p* < 0.05;

*** *p* < 0.001;

† *p* < 0.10 (marginal significance)
